# Can we reposition finite element human body model like dummies?

**DOI:** 10.3389/fbioe.2023.1176818

**Published:** 2023-05-17

**Authors:** Jisi Tang, Qing Zhou, Wenxuan Shen, Wentao Chen, Puyuan Tan

**Affiliations:** State Key Laboratory of Automotive Safety and Energy, School of Vehicle and Mobility, Tsinghua University, Beijing, China

**Keywords:** human body model, repositioning, vehicle safety, finite element analysis, kinesiology

## Abstract

Rapidly repositioning finite element human body models (FE-HBMs) with high biofidelity is an important but notorious problem in vehicle safety and injury biomechanics. We propose to reposition the FE-HBM in a dummy-like manner, i.e., through pose parameters prescribing joint configurations. Skeletons are reconfigured along the trajectories inferred from model-specific bone geometries. We leverage differential geometry to steer equidistant moves along the congruent articulated bone surfaces. Soft tissues are subsequently adapted to reconfigured skeletons through a series of operations. The morph–contact algorithm allows the joint capsule to slide and wrap around the repositioned skeletons. Nodes on the deformed capsule are redistributed following an optimization-based approach to enhance element regularity. The soft tissues are transformed accordingly via thin plate spline. The proposed toolbox can reposition the Total Human Body Model for Safety (THUMS) in a few minutes on a whole-body level. The repositioned models are simulation-ready, with mesh quality maintained on a comparable level to the baseline. Simulations of car-to-pedestrian impact with repositioned models exhibiting active collision-avoidance maneuvers are demonstrated to illustrate the efficacy of our method. This study offers an intuitive, effective, and efficient way to reposition FE-HBMs. It benefits all posture-sensitive works, e.g., out-of-position occupant safety and adaptive pedestrian protection. Pose parameters, as an intermediate representation, join our method with recently prosperous perception and reconstruction techniques of the human body. In the future, it is promising to build a high-fidelity digital twin of real-world accidents using the proposed method and investigate human biomechanics therein, which is of profound significance in reshaping transportation safety studies in the future.

## 1 Introduction

Finite element human body models (FE-HBMs), e.g., Total Human Body Model for Safety (THUMS) (Iwamoto et al., 2002) and Global Human Body Model Consortium (GHBMC) (Gayzik et al., 2011) models, are widely exploited to evaluate the biomechanical response of the human body. These models are normally only available in standard occupant and pedestrian postures, i.e., upright seated, standing, and mid-stance walking. However, in the field of transportation safety, the body can exhibit various non-standard postures, e.g., walking, running, or jogging for pedestrians (Chidester and Isenberg, 2001) and reclined (Reed et al., 2019) or other out-of-position postures (McMurry et al., 2018) for occupants. Conclusions from a few representative postural configurations cannot be extended to general cases in a scientifically trustful way because our developed understandings and vehicle safety designs accordingly might be biased in this sense. It is hence urgent to establish a standard, systematic framework to adapt the canonical baseline models to general configurations, to deepen and reinforce our insights into the postural effect on human safety on roads.

A great merit of the FE-HBM is the superior biofidelity, which, on the contrary, also limits the model’s usability due to subject specificity and anatomical complexity. Despite the enormous demands from academic and industrial activities, there are very few theoretical studies on how to reposition a modern FE-HBM with a sophisticated hierarchy of anatomy.

The existing works present a paradigm shift. In the early years, pre-simulations were commonly used (Tang et al., 2016). External forces are directly or indirectly, e.g., via strings like a marionette, applied to limbs or other distal components to reconfigure the model. There are several drawbacks to this process. The simulation lasts an exceptionally long time to reach equilibrium. The repositioned models usually report degraded mesh quality and rely on manual corrections thereafter to rectify the distortions. Besides, a fundamental-level problem is that the resulting kinematics is not guaranteed to be anatomically correct (Jani et al., 2009) because the HBM lacks part of the necessary kinematical constraints from connective tissues. Researchers have turned to kinesiology for answers. Kinesiology generally relates anatomy, geometry, and physiology to joint kinematics. For example, Blankevoort et al. (1990) associated knee flexion to the transepicondylar axis (TEA), which can be inferred from bone geometry. Such knowledge offers explicit control of bone movement in simulation.

Kinesiology-driven strategy inspires researchers as it is feasible to reconfigure skeletons with rigid transformation, adapt soft tissues by relocating the associated contours (Jani et al., 2009), or perform mesh smoothing via the kriging method (Desai et al., 2012). These approaches are simulation free and significantly accelerate the computation. PIPER implements the methods on the latest THUMS and GHBMC models and formulates an integrated solution for engineering use (Chhabra et al., 2017). Another simulation-free solution is to simplify the HBM by approximating joint kinematics with structurally similar hinges and rigidifying the deformable parts, like the fast GHBMC model (Schwartz et al., 2015). The simplified model can be easily repositioned in FE preprocessors like dummies. However, the deteriorated biofidelity in simplification makes the approach unsuitable for fine-level tasks.

There are two major issues remaining. First, kinesiology studies are as yet far from completion. The current conclusions are founded on reverse engineering; take the most complicated and widely investigated knee movement as an instance. Existing studies regress the experimentally measured motion to certain anatomical axes, e.g., the transepicondylar axis (Blankevoort et al., 1990) and geometric center axes (Asano et al., 2001), but knee flexion cannot be globally parameterized about any fixed axis as the condyle shape is highly irregular. It implies that forward computation considering subject-specific details is essential. Second, the current morphing or mesh smoothing methods were originally developed and tested on early HBMs. Today’s models are more sophisticated in anatomy. The current methods have to be updated to well handle finer meshes and more details.

In this study, we propose to reposition the FE-HBM in a fast and biofidelic way. The method is simulation free, relying on a combination of geometry-informed transformation, interpolation, and optimization. Joint trajectories are derived from model-specific geometries. At the tissue level, our method leverages a thin plate spline to adapt soft tissues to the reconfigured skeletons. A series of operations are developed to tackle the error-prone sliding motion at the interfaces. In subsequent contexts, we will introduce the basic idea of the approach, derivation of arthrokinematics, and emulation of tissue deformation in sequence. Efficacy and robustness of the method are demonstrated by constructing pedestrian models exhibiting unusual collision-avoidance poses. Significance, theories, and limitations of the method are then discussed. Finally, we prospect the impact to relevant posture-sensitive topics in the future.

## 2 General idea, kinesiology, and computational implementation of repositioning FE-HBM

**FIGURE 1:**
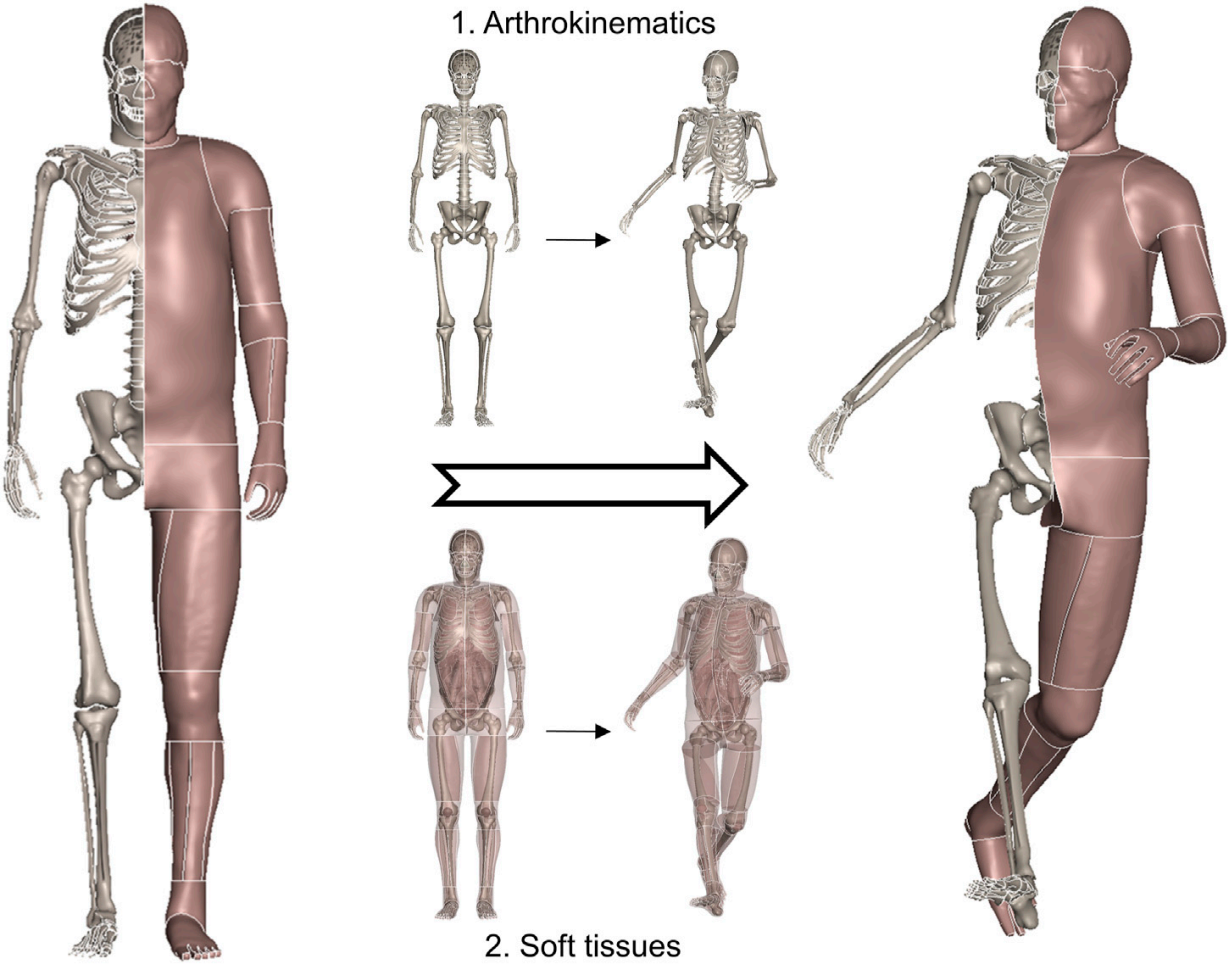
General schematic demonstrating a physical posture change of the human body. The bones are first repositioned following anatomically correct arthrokinematics, i.e., joint trajectory, and soft tissues are deformed accordingly.

We start by elaborating on the general idea of this study. The human body is anatomically composed of rigid skeletal parts and deformable soft tissues. Posture, also referred to as pose, describes the physical configuration of the human body. When executing a specific posture change (Figure 1), the bones are first repositioned according to a group of joint parameters, and then the soft tissues are adapted accordingly. As introduced, current studies have reached a consensus that bones can be reconfigured via rigid transformation, and soft tissues are deformed through non-physical emulation, such as interpolation, instead of time-consuming simulations. This study follows the mainstream pipeline and focuses on addressing the reported challenging issues of computational implementation on sophisticated modern HBMs.

Joints are transformable connective mechanisms. Pose parameters define the physical configuration of joints. Dummies can be conveniently repositioned, as the hinge structure mechanically prescribes inter-component kinematics. When it comes to the FE-HBM, it replicates only a specific configuration of the human body of interest. Human joints are not hinge type. Movements are guided and constrained by face-to-face articular contact, so there are no structural axes to intuitively leverage on. A crucial point in repositioning is to let the model move in an anatomy-compatible way. Conventional studies approximate the movements with simple hinge-type motion, e.g., rotation about a fixed axis. Nonetheless, the equivalent helical axes in realistic human movements are constantly varying from one configuration to another. We propose to derive subject-specific joint trajectories from congruent bone geometries. It allows the estimated trajectory to always be tangential to the shapes in articulation. The entire trajectories naturally parameterize human movements and make the skeleton reconfigurable.

We introduce a method to rapidly emulate soft-tissue deformation. Our approach leverages thin plate spline, a specific type of kernel-based interpolation, to generate a displacement field that is prescribed for bone movements. Different from conventional studies, our method considers the sliding boundary condition, that is, many interacting instances in a realistic human body slide along each other in movement. Classical interpolation cannot tackle sliding-induced discontinuity in displacement. Therefore, we develop a morph–contact algorithm, where morph takes charge of continuous deformation and contact eliminates penetrations in a pseudo-physical way. In addition, we develop an optimization-based refinement to ensure the interface meshes are regular as the baseline.

Finally, we introduce a systematic technical framework founded on the aforementioned algorithms to reposition the FE-HBM of interest in a way that it is efficient as a dummy and biofidelic as the realistic human body.

## 3 Geometry-informed parameterization of joint motion

In this study, we consider the shoulder, elbow, hip, knee, and thoracic and lumbar spine. Motion at these joints is parameterized based on model-specific geometries. In general, flexion/extension, adduction/abduction, and internal/external rotation are the degrees of freedom (DOFs) of interest, while at the knee, elbow, and thoracic spine, we only focus on flexion due to the anatomically limited motion range.

### 3.1 Knee

The knee is a compound joint. It consists of the medial and lateral compartments of the tibiofemoral and patellofemoral joints. In orthopedics and kinesiology, it is commonly believed that knee motion is 2-DOF: flexion/extension (primary) and slight internal/external rotation. However, experimental studies have advocated that for an individual, all secondary DOFs are coupled to the primary motion. That is, the trajectory can be fully described by a single parameter, but the parameter does not coincide with any off-the-shelf DOF following existing decomposition schemes (Grood and Suntay, 1983).

**FIGURE 2:**
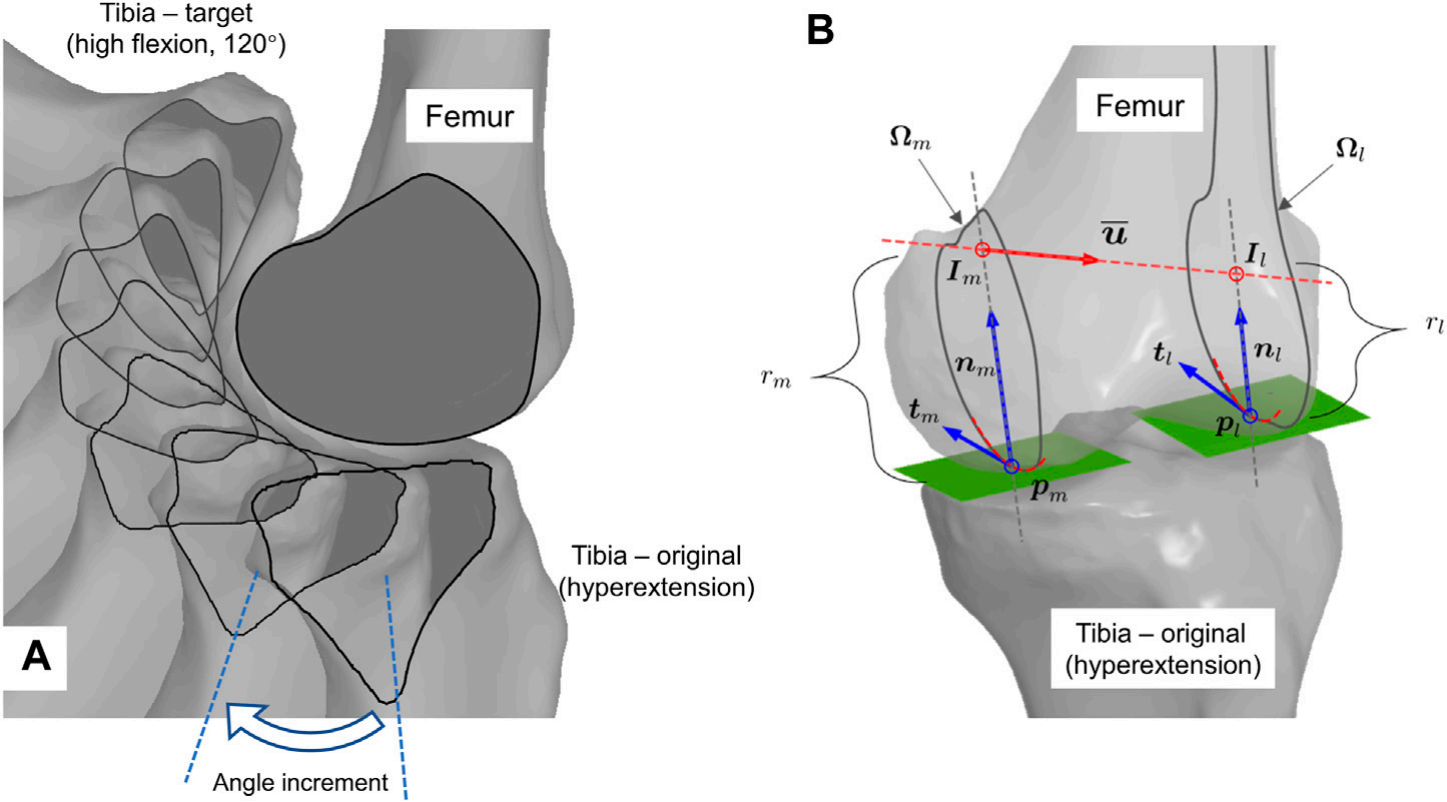
Geometry-informed computation of knee flexion. **(A)** Graphical illustration of iterative flexion estimation. Tibia is incrementally rotated about helical axis inferred from current bone configuration. **(B)** Computation scheme of geometry-compatible tibial rotation, i.e., the instant helical axis.

We propose a geometry-based estimation algorithm for knee flexion (Tang et al., 2017). The integrated trajectory is always equidistant to the femoral condyle at both medial and lateral compartments (Figure 2A shows the medial part). The entire tibiofemoral trajectory is incrementally constructed by concatenating piecewise rotations about the instantaneous helical axis (Figure 2A), where computation of the axis according to the current bone configuration is the key point (Figure 2B).

The helical axis approximates the first- and second-order derivatives of the trajectory. The computation in Figure 2A is as follows: first, in Figure 2B, the contact points on the condyle, $p_{m}$ and $p_{l}$ , are found by searching for a pair of points with collinear normal with the corresponding tibial plateau, $n_{m}$ and $n_{l}$ (Tang et al., 2017). Second, the instant flexion axis, $\bar{u}$ in Figure 2B, is inferred from the current configuration, following a series of mathematical operations, which are updated in this study and will be introduced hereafter in detail. Third, the tibia is rotated about the axis with an infinitesimal or finite angle. Finally, steps 1–3 are repeated until the target configuration is reached (the repeated contours in Figure 2A).

There are two issues that have not been addressed in the pilot study. One is that the geometrically compatible trajectory in each step is not unique, but the pilot study gives only one heuristic solution. The second is that the computation strictly demands a quadrilateral mesh, but the triangular mesh is more common and general in computer-aided design (CAD) environments. We tackle them one by one in this work.

We propose to compute the helical axis ( $\bar{u}$ in Figure 2B) from the perspective of *differential geometry*. Based on clinical and kinesiological observations, the femoral condyle and tibial plateau are constantly in contact during motion. Geometrically, being in contact implies that the motion is locally tangential. Instant rotation about the helical axis passing through both normal vectors ( $n_{m}$ and $n_{l}$ ) on the medial and lateral condyles naturally satisfies the tangent condition. The condition therefore constructs a 2-dimensional space, parameterized by $r_{m}$ and $r_{l}$ , measuring how long the intersection points ( $I_{m}$ and $I_{l}$ ) deviate from the corresponding contact points on the condyle.

Every combination of $r_{m}$ and $r_{l}$ draws a line, and rotation about the line is locally tangential to the tibia. We therefore introduce a geometrically “neutral” path among the feasible solutions. From a geometric point of view, if rotation along the helical axis coincides with the associated osculating circle (red dotted arcs in Figure 2B) derived from the normal curvature of the intersecting contour curves ( $\Omega_{m}$ and $\Omega_{l}$ ), the tibia makes contact with the femur at the same points after an infinitesimal rotation. In differential geometry, *normal curvature* is a generalization of the curvature to a 2-manifold, which is the curvature of the curve projected onto the plane spanned by the normal and associate tangents (see textbooks on differential geometry for more mathematical details if interested). The contact point is static following the derived motion if viewed from the tibia. The trajectory concatenating such rotations, as demonstrated in Figure 2A, is the geometrically “neutral” path. Here, “geometrically” implies that the path is fully geometry dependent, while “neutral” implies to the stationary condition of the contact point on the tibia. The geometrically neutral path gives a rigorous definition of the heuristic solution given in a previous study (Tang et al., 2017). As introduced, the geometrically allowed motions construct a 2-dimensional subspace, and the geometrically neutral path is essentially the origin of the space in each iteration. In practice, there might be oscillations in the path due to flexibility of the connective tissues, which can be characterized by measuring the trace of the contact point on the tibial plateau in an experiment. The measured deviations of the contact points can be computationally retrieved by shifting the intersection points ( $I_{m}$ and $I_{l}$ ) accordingly. The resulting path is not “neutral” anymore thereafter. In a word, the neutral path is fully geometry dependent, and the realistic movement can be reconstructed on this baseline with additional knowledge or measurements.

The definition of the neutral path is also beneficial in enhancing joint stability. Anatomically, wedge-sectioned, crescent moon-shaped meniscus supports femur-to-tibia contact radially inward, centered at the contact point. The static center indicates that the meniscus can stay at its original position on the plateau to stabilize the articulation without having to deform or slide. Mechanically, load transfer is more stable through the slightly concave central part of the tibial plateau than the inclined peripheral section, such that a static contact near the center is more stable as well.

In computation, all shapes are represented by triangle meshes. Femoral condyles are preserved in the original convex shape, whereas tibia plateau surfaces are approximated as planes such as how Wilson et al. (1998) did. Calculating the radius of the osculating circle is crucial for figuring out the neutral path. There is a technical obstacle in that curvature is a second-order differential in its theoretical definition, but mesh is a discrete data structure, not naturally differentiable. Here, we refer to the normal cycle theory–based method proposed by Cohen-Steiner and Morvan (2003). It gives an elegant form to estimate per-vertex curvature tensor C on triangular mesh $\left(V,E,F\right)$ with exterior calculus, as shown below.

$$C\left(p\right)=\frac{1}{\left|A\left(p\right)\right|}\underset{e\in A\left(p\right)}{\sum }\beta \left(e\right)\left|e\cap A\left(p\right)\right|\bar{e}⊗\bar{e},$$

where e is an edge incidental to vertex p ; $\beta \left(e\right)$ is the signed dihedral angle between faces incidental at edge e , which is positive if convex; $A\left(p\right)$ is the local neighborhood around vertex p with $\left|A\left(p\right)\right|$ as its area; $\left|e\cap A\left(p\right)\right|$ is the length of the part on edge e that falls within $A\left(p\right)$ ; $\bar{e}$ is the length-normalized edge vector; and ⊗ means tensor product. In practice, $A\left(p\right)$ is usually the 1-ring neighborhood, so $\left|A\left(p\right)\right|$ is the Voronoi area. We precompute curvature tensor at all vertices on the medical and lateral femoral condyle surfaces. For a general point inside a triangle, the tensor could be computed with a weighted sum based on barycentric coordinates.

Given a tangential direction of interest at vertex p , normal curvature $\kappa_{n}$ can be computed with curvature tensor C and the associated tangent vector t via a quadratic form as

$$\kappa_{n}=t^{T}\cdot C\left(p\right)\cdot t.$$

Given the current femur and tibia configuration, with contact points $p_{m}$ and $p_{l}$ as well as condyle normal vectors $n_{m}$ and $n_{l}$ provided, we optimize $r_{m}$ and $r_{l}$ to align with the derived intersection points $I_{m}$ and $I_{l}$ , with the center of the osculating curves computed from the normal curvatures on both condyles, using the equation

$$\left(r_{m},r_{l}\right)=\underset{r_{m},r_{l}}{\arg\;\min}\left({\left(r_{m}-\frac{1}{t_{m}^{T}\cdot C\left(p_{m}\right)\cdot t_{m}}\right)}^{2}+{\left(r_{l}-\frac{1}{t_{l}^{T}\cdot C\left(p_{l}\right)\cdot t_{l}}\right)}^{2}\right),$$

where $C\left(p_{m}\right)$ and $C\left(p_{l}\right)$ are curvature tensors at the contact points, on the medial and lateral condyles, respectively, and $t_{m}$ and $t_{l}$ are tangent vectors perpendicular to the flexion axis corresponding to current $r_{m}$ and $r_{l}$ . The $t^{T}\cdot C\left(p\right)\cdot t$ terms compute the corresponding normal curvature, and the reciprocal terms compute nominal radii reciprocal to the normal curvatures. The radii are supposed to equal the desired parameters for optimization, such that we optimize the mean square error between them and $r_{m}$ and $r_{l}$ .

The intersection points $I_{m}$ and $I_{l}$ can be intuitively got by deflecting contact points with the computed radii, and the instant flexion axis $\bar{u}$ joins $I_{m}$ and $I_{l}$ . Given a specific femur and tibia configuration, we can always compute the instant axis following the abovementioned steps. The tibia can be iteratively rotated about the current axis with an incremental angle step, e.g., 0.5° as an empirical suggestion, until the tibia reaches the desired flexion position. For example, the computation can stop at any position per the user’s request (Figure 2A).

### 3.2 Other synovial joints

Besides the knee, we also parameterize motion at other major synovial joints, i.e., the shoulder, elbow, and hip. These joints are also articulation guided, so the motions are derived in a similar way to that done for the knee. Fortunately, when compared to the knee, their anatomies are simpler and the surface shapes are more regular as well. We derive the motions with global parameterization by approximating the joint anatomy with structurally similar hinges.

**FIGURE 3:**
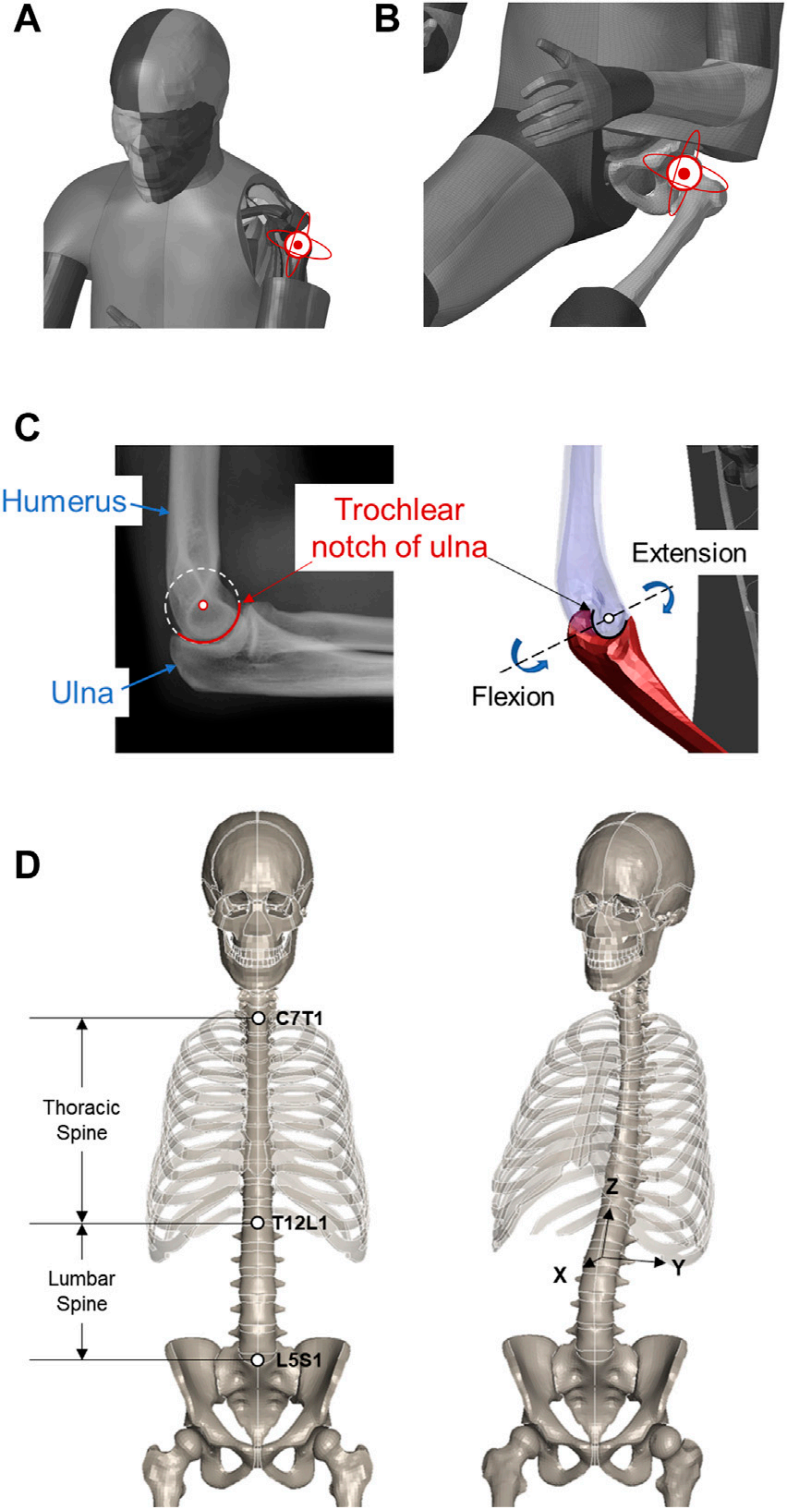
Motion DOFs at major movable joints. **(A)** Shoulder. **(B)** Hip. **(C)** Elbow. **(D)** Spine.

At the shoulder and hip, the glenohumeral and acetabulofemoral joints are ball-and-socket type, so the associated arthrokinematics is characterized by 3D rotation about the center of the humerus and femur heads, respectively (Figures 3A, B), where the centers are estimated from the nodal coordinates on the sphere-like surfaces in a least squares manner. Upon implementation, we use a group of intrinsic Euler angles in the internal/external, flexion/extension, and adduction/abduction sequences to parameterize the 3D rotation.

At the elbow, we consider the hinge-type humeroulnar joint. The flexion/extension motion is represented by an in-plane rotation about the estimated center of the trochlear notch of the ulna (Figure 3C), similar to the least squares manner. In the current study, supination and pronation at the proximal radioulnar joint are not applied, and the radius moves with the ulna accordingly. Like the knee, the elbow only takes a flexion angle as input.

### 3.3 Spine

The spine, also known as the *vertebral column*, consists of the entire set of vertebrae. A total of 33 vertebral bony segments, i.e., the *vertebrae*, are normally divided into five regions, seven *cervical*, twelve *thoracic*, five *lumbar*, five *sacral*, and four *coccygeal segments*. Each region has its own morphology that reflects its specific function and pattern of movement. In kinesiology, the entire spine pose is usually described per segment in a 3D rotation-like way, i.e., with Euler angles. This abstracts the base-to-end pose of each segment on a high level but cannot intuitively specify the spatial configuration of each vertebra along the path.

When repositioning the FE-HBM, we focus on flexion of the thoracic and 3D rotation of the lumbar segments (Figure 3D). A key task in practice is to retrieve the kinematics of individual vertebrae from the given abstract angles. Referring to Desai et al. (2012), we manually define the rotation center for each vertebra by averaging the center of the top and bottom faces. The overall rotation angles are evenly split to every vertebra. Rotation matrices are generated accordingly following the Rodrigues formula. The involved vertebrae are then transformed about their own center.

## 4 Emulation of soft-tissue deformation

We propose to emulate, instead of simulate, the deformation of the involved soft tissues according to the bone movements. The difference is, emulation is not strictly based on the real-world physics, but simulation is. Conventional simulation-based approaches are time consuming and prone to severely distorting elements. Emulation is implemented to accelerate the computation while intuitively controlling the pattern of nodal displacements. The mechanism of emulation can be roughly divided into two parts. Continuous transformations, e.g., flesh deformation driven by bone movements, are approximated via interpolation, while in the case of where discontinuous transformations occur, an innovation is to introduce a morph–contact algorithm to incorporate light physics to tackle the singular patterns.

### 4.1 Mesh morphing and thin plate spline

Mesh morphing methods have been extensively studied and implemented in tuning the morphology of HBMs to account for anthropometric variations in the population (Tang et al., 2020; Hu et al., 2019a). Mesh morphing relies on a few prescribed correspondences to transform a shape into target geometry. A group of homologous landmarks are selected on both the source and target shapes. The landmarks are exactly one-to-one corresponded in a much less number than the entire nodes. Sparse correspondences are therefore anchored between the shapes to associate. An interpolation function is a continuous function defined on the source space, while at landmark positions, it outputs the corresponding landmarks on the target shape and gives a smooth transition between them by minimizing specific kinds of energy.

Thin plate spline (TPS) is a commonly exploited interpolation function inspired by minimizing the internal energy of bending thin plates. Please refer to the study by Hwang et al. (2016) for mathematical derivation about TPS and its implementation in detail if interested.

Adapting soft tissues to repositioned skeletons can follow the same pipeline. Nodes on the bones are natural anchors. The source and target landmarks are assigned as nodal coordinates before and after transformation, respectively. The soft-tissue nodes can be repositioned accordingly.

### 4.2 Morph–contact algorithm, refinement, and implementation

A long-term challenging problem in conventional interpolation-based methods is the sliding interface. When going across the boundary, there is a sudden jump in nodal displacements, therefore sliding “lacerates” the displacement field. This leads to discontinuity in the function of interest. The spline function approximates the target function by regressing coefficients with respect to pre-defined continuous bases, so the sliding-induced discontinuity cannot be sufficiently reconstructed in this sense. Especially at the synovial joints, such as the shoulder, elbow, hip, and knee, bone segments that originally contact with the capsule might move into the joint space. If intuitively interpolated by bone landmarks, the flesh becomes overstretched and this will lead to severe element distortion.

**FIGURE 4:**
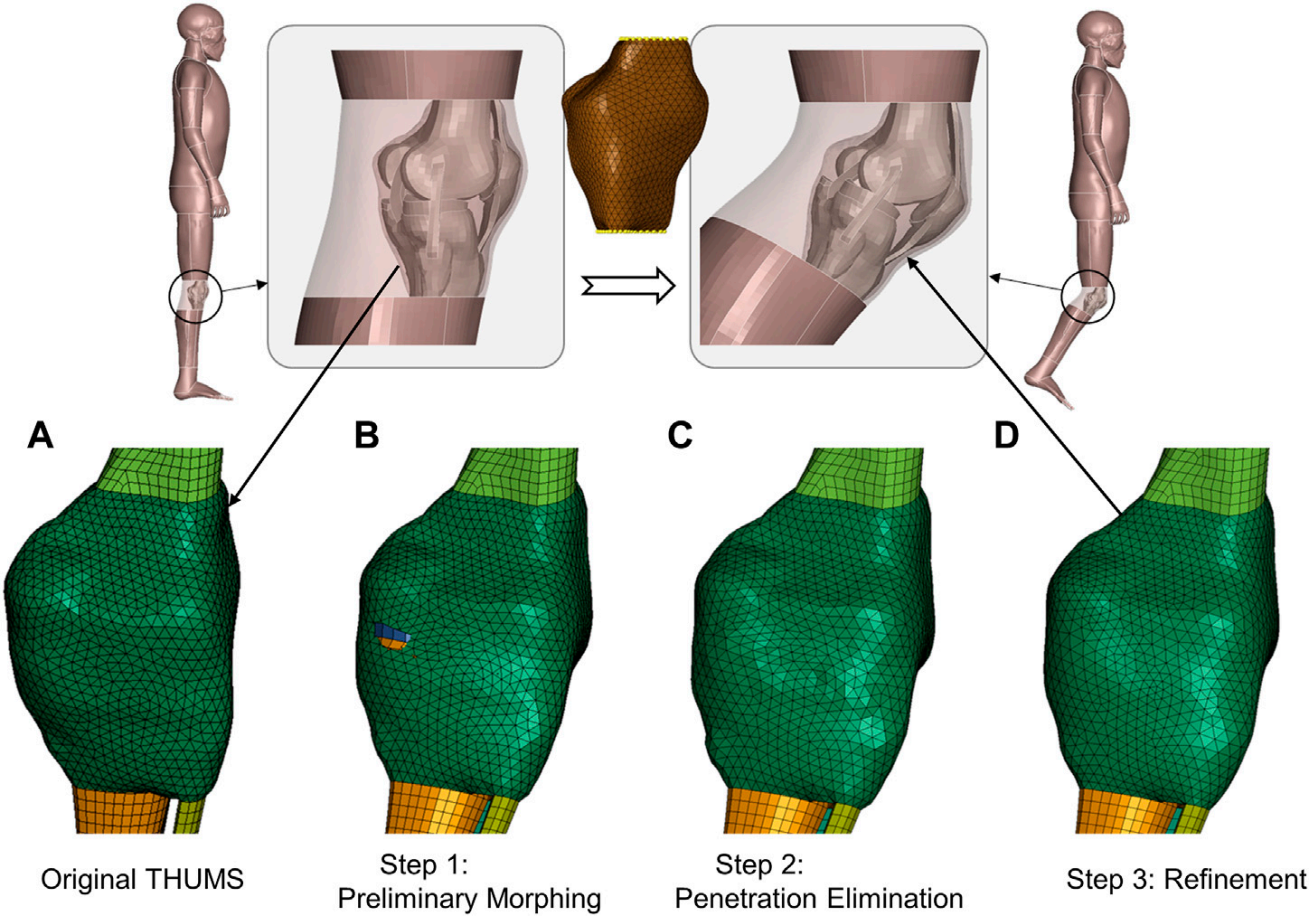
Schematic of emulation of soft-tissue deformation given prescribed bone movements. A 40-degree right knee flexion is demonstrated via visualizing transformation patterns of the knee capsule after each processing step. The transparent part depicts the flesh bound by the capsule, and upper and lower legs. The yellow dots in the middle subfigure are landmarks for preliminary deforming. **(A)** Original capsule mesh. **(B)** After a mesh morphing with a reduced set of landmarks, the capsule is preliminarily deformed, but there are obvious penetrations. **(C)** After the penetrations are detected and eliminated through node deflections, the mesh regularity is partly deteriorated, and there are local oscillations around the pulled nodes. **(D)** After refinement, the mesh regular is the same as the baseline, which promotes high-quality transformation of the flesh of interest.

We take the knee of the THUMS model as an instance to elaborate our method (Figure 4). The contour of the wrapping capsule component is divided into four segments. The proximal and distal faces are attached to the underlying skeletal parts, i.e., the femur and tibia, respectively. They rigidly move with the bones. The interior face is the segment of interest, which slides along the bone–flesh interface. We propose a morph–contact strategy that jointly satisfies the geometric constraints and preserves high mesh quality. The remaining nodes are subsequently transformed via thin plate spline, following the pattern prescribed by the other three segments.

The key point is to tackle the sliding-induced discontinuity. We suggest manufacturing the original capsule contour (Figure 4A) via light physics–based emulation. The contour is first preliminarily interpolated according to a reduced set of bone landmarks. The transformed interface roughly approximates the displacement field in space, but there are penetrations around the joint space (Figure 4B). These penetrations are detected by a customized step inspired by contact algorithms in computational mechanics. In brief, the program computes the signed distance of bone nodes involved (slave nodes in classical contact detection algorithm) to the capsule surface (master surface) and finds the nodes experiencing orientation flips. The surface patches associated with the “penetrated” nodes are pulled outward along the surface normal until the penetrations are eliminated (Figure 4C).

The interior surface wraps bones with a physically reasonable contour after the above steps, but the nodes on the surface are not guaranteed to be regularly positioned and are usually oscillatory. This leads to overstretched or distorted elements. This method relies on an optimization-based refinement to regularize the mesh pattern. The involved nodes are redistributed to simultaneously 1) pertain to the original shape, 2) penalize inconsistency of edge length, and 3) ensure coherence of normal vectors. A well-established implementation is already available in PyTorch3d (Johnson et al., 2020), a Python library built upon PyTorch (Paszke et al., 2019) for geometric deep learning, where the Chamfer distance is used to evaluate how much the surfaces of interest deviate from each other. Figure 4D displays the refined capsule interface, and the mesh regularity is significantly improved when compared to the raw penetration-fixed mesh in Figure 4C.

The refined interior capsule interface, in company with the interfaces interacting with the static and moving bones, constructs the boundary landmarks. We can intuitively emulate soft-tissue deformation (the transparent parts in Figure 4) via TPS and gain high-quality solid elements thereafter.

Soft tissues surrounding other movable joints that are discussed in Section 3 are processed following the aforementioned steps. At the shoulder, hip, and elbow, there are sliding interfaces as well, so the morph–contact algorithm is applied. Tissues attached to the spine are simply interpolated via TPS.

## 5 Fast and biofidelic repositioning toolbox

### 5.1 Framework

We formulate the fast and biofidelic toolbox for FE-HBM repositioning via integrating the algorithms of Section 3 and Section 4. The toolbox takes joint parameters as input, i.e., 3 × 3 rotation matrices for shoulder and hip joints, flexion angles for elbow and knee joints, as well as thoracic and lumbar rotations. The center of the humerus head, trochlear notch of the ulna and femur heads, and trajectory of knee flexion are precomputed based on model geometry. In practice, the pose parameters are achievable from various sources, such as computer vision, computer graphics, orthopedics, and ergonomics.

### 5.2 Demos

We apply the proposed toolbox to THUMS occupant and pedestrian models (AM50, v4.1/4.0.2) to demonstrate its efficacy and availability in injury biomechanics studies. Figure 1 displays the THUMS model repositioned into a collision-avoidance pose. The limbs are sufficiently stretched compared to the baseline, so the case is challenging enough to tackle with local, large deformations. Jacobian, warpage, and aspect ratio are selected as the evaluation metrics of the mesh quality. Among the concerned 1,527,480 3D elements, 26,275 (1.7%), 5,166 (0.3%), and 4,836 (0.3%) elements do not satisfy the checking criteria for the baseline model in the sequence of warpage (>15.0), aspect ratio (>5.0), and Jacobian (<0.5); the corresponding values for our repositioned model in Figure 1 are 26,699 (1.8%), 5,437 (0.3%), and 4,943 (0.3%). The increase in the number of bad-quality elements is not significant, which advocates that our method does not deteriorate the 3D elements and well preserves the regularity of the initial mesh.

**FIGURE 5:**
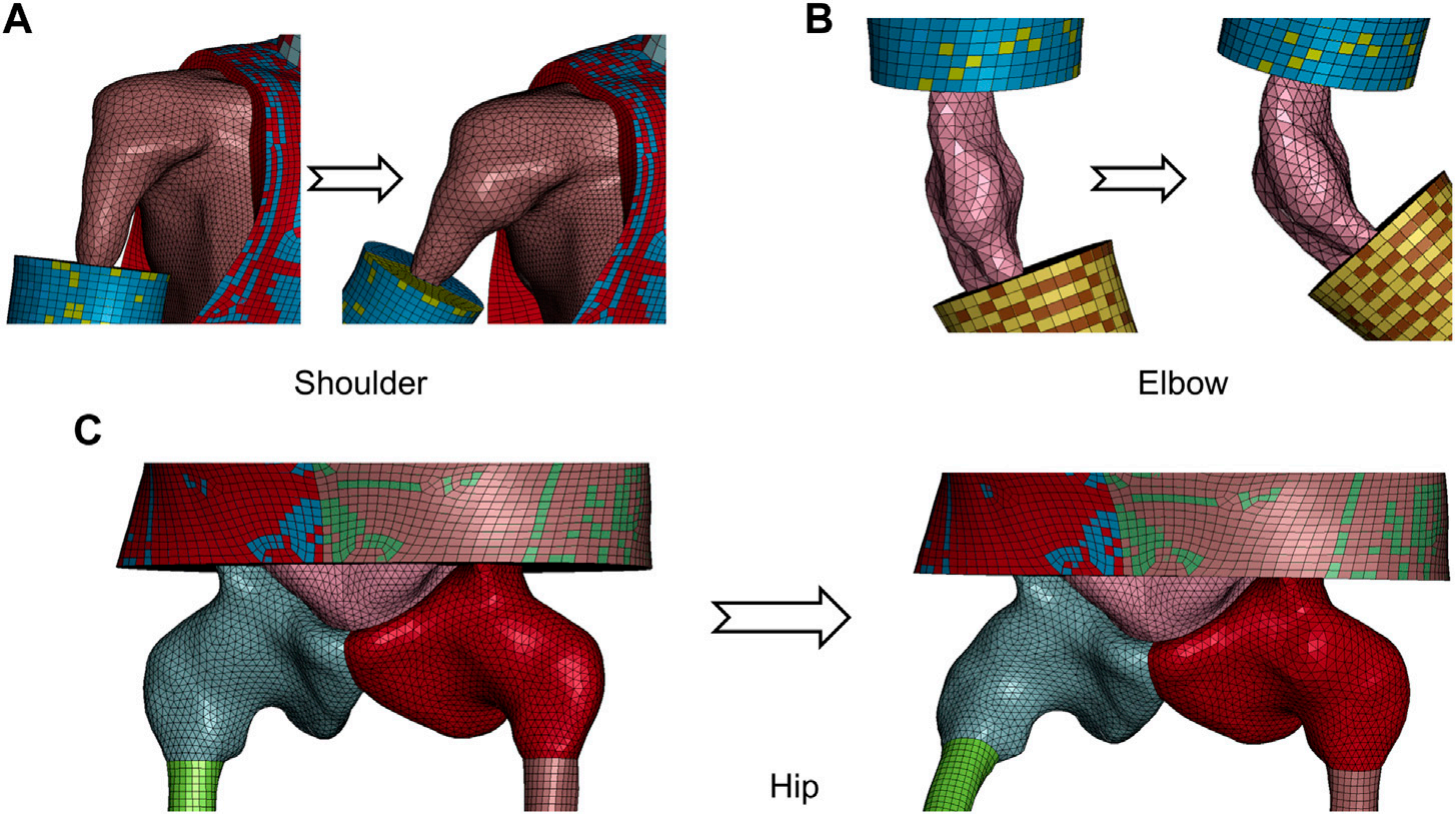
Visualization of transformed capsule surface mesh of major synovial joints from repositioned model in Figure 1. **(A)** Shoulder. **(B)** Elbow. **(C)** Hip.

Besides the knee interior, i.e., joint capsule surface, demonstrated in Figure 4, Figure 5 visualizes the transformed interior of other synovial joints, i.e., the shoulder, elbow, and hip. The transformation following the steps introduced in the previous section sufficiently preserves the mesh pattern of the bone–flesh interfaces and smoothly completes shape deformation. Regular interior mesh theoretically guarantees the mesh quality of the emulated flesh mesh.

**FIGURE 6:**
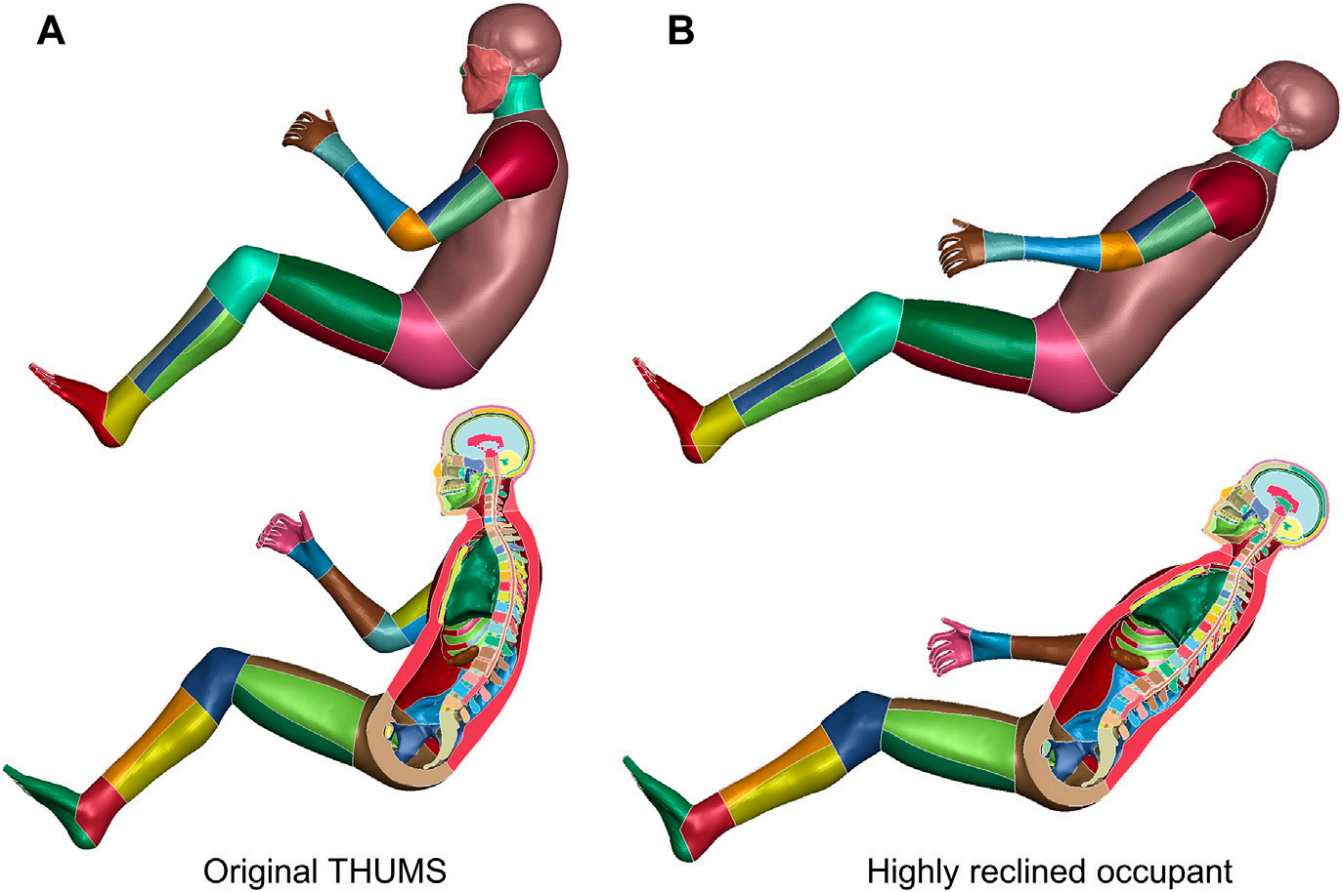
Reclined THUMS occupant model. The original upright seated THUMS occupant model **(A)** is repositioned to a highly reclined posture **(B)** with our method.

The occupant model is repositioned to a highly reclined pose, according to volunteer tests documented by Reed et al. (2019). Anatomical landmarks of representative vertebral centers are prescribed. The THUMS occupant model is flexibly reclined (Figure 6). Besides, the forearms are laid down and the thighs are extended to better mimic a relaxed reclined pose.

**FIGURE 7:**
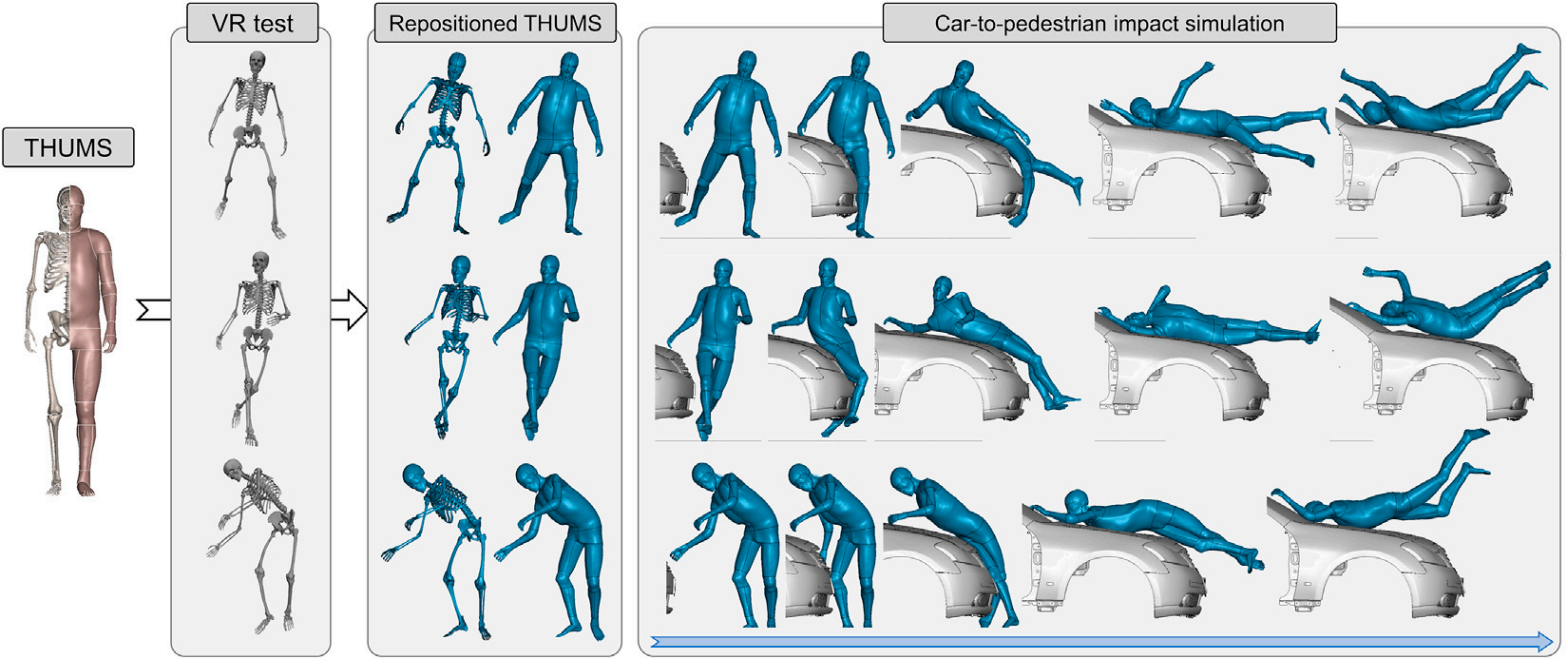
Demonstration of the virtual reality–physical simulation pipeline. It is convenient to convert baseline THUMS pedestrian model into any arbitrary pose captured by the MoCap system (documented in Li et al., 2021; data visualized in OpenSim) with our method. The repositioned models are robust when exposed to 40 km/h impact from vehicle.

In terms of pedestrians, Nie et al. (2021) established a virtual reality–based (VR) platform to investigate a pedestrian’s active avoidance maneuver against imminent car crashes (Li et al., 2021). The instantaneous 3D poses of the volunteers are captured by an optical motion capture system (MoCap). In Figure 7, we demonstrate three repositioned models. These models are ready for simulation, with comparable mesh quality as the baseline. The car-to-pedestrian simulation results suggest that diverse avoidance maneuvers lead to significantly varying impact responses.

**FIGURE 8:**
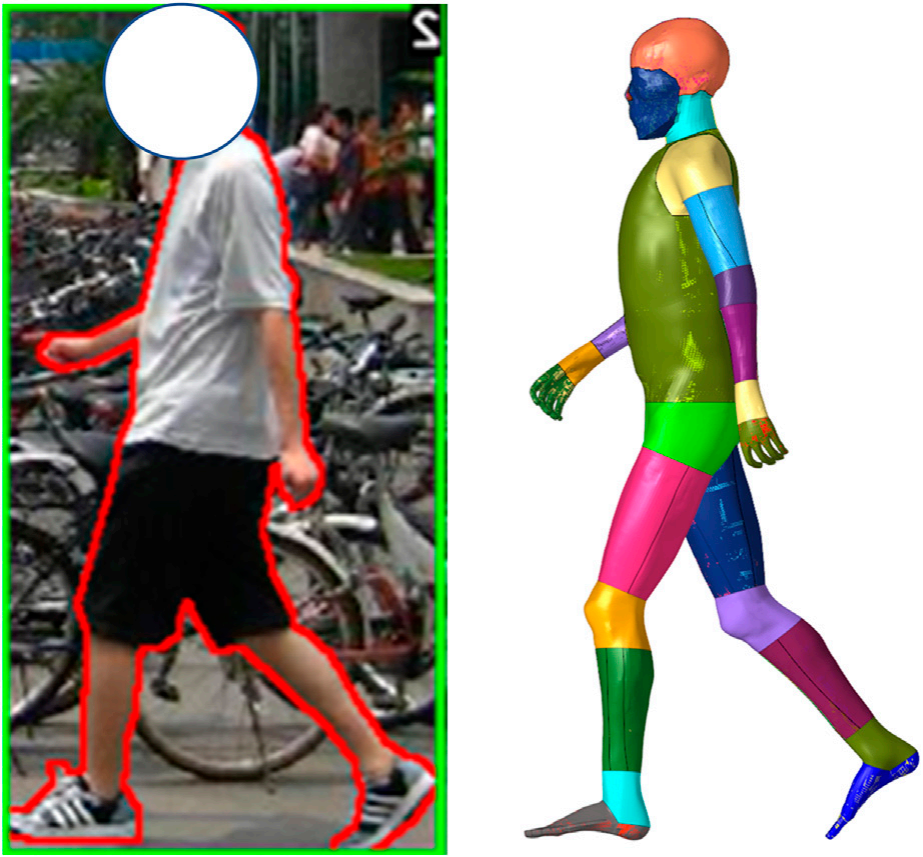
Pose-specific pedestrian model. The data source is a plain image depicting a walking individual. Pose parameters are inferred by Expose (Choutas et al., 2020), a neural network–based pose estimation model. The baseline THUMS model is then repositioned to the recognized posture.

Laboratory experiments can gather sequential 3D pose data, but these tests are very limited. Most accidents on roads are recorded by onboard or roadside cameras. Computer vision models can recognize human poses from images, e.g., OpenPose (Cao et al., 2017), and its variants (Tang and Zhou, 2021). We exploit Expose (Choutas et al., 2020) in this study, to infer pedestrian poses in an open-source data set (Figure 8). Expose is founded on SMPL (Loper et al., 2015), which is a parametric human shape model, so the inferred pose parameters follow the SMPL convention, and we adapt them to the presented format.

In practice, inference with Expose takes 20 s, and the toolbox takes about 500 s on a desktop PC with Intel 10700 CPU and NVIDIA RTX 3070 Ti GPU. The entire pipeline is fully automatic, and the generated model is ready to use, with comparable mesh quality to the baselines without any post-processing.

## 6 Discussion

### 6.1 Significance

In this study, we establish a fast and biofidelic repositioning method for the finite element human body model. It takes joint parameters as input, like conventional dummy models, to reconfigure the skeleton along geometrically embedded trajectories. It also deforms soft tissues via a hybrid transformation method that combines interpolation and optimization in a simulation-free manner. The proposed toolbox is compatible with multimodality pose representations from a variety of sensors and perception technologies, e.g., pedestrian recognition via computer vision (Tang and Zhou, 2021). The transformation is time efficient and highly biofidelic. Generated models are simulation ready, without demands on manual editing, smoothing, or other kinds of post-processing. Our method grants the FE-HBM comparable usability to dummies, such that studies or developments conventionally performed with a dummy can be easily transferred, e.g., restraint system development and safety evaluation/assessment.

The demonstrated virtual reality–physical simulation pipeline (Figure 6) has profound significance. It forms a new paradigm for studies on musculoskeletal biomechanics. The original human–system interaction in virtual reality is one way. Volunteers visually perceive imminent car crashes by the eye and act physically to avoid them (Li et al., 2021), but they will not be really hit if the attempts fail, so the message in passing is only virtual to physical. This study, instead, establishes a two-way interaction. After the virtual-to-physical perception, there is a physical-to-virtual response. The time-sensitive human reaction can intuitively lead to diversified impact responses (Figure 6). In other words, our method grants the virtual reality space with physical principles, e.g., Newton’s law.

In the long term, the pipeline promotes digital twin studies. Take the promising integrated safety technology as an instance. At an instant prior to a safety-critical event, we can construct a subject, scenario, and reaction-specific HBM. Given an adaptable countermeasure system, e.g., an adaptive bumper (Shen et al., 2022), the system can optimize for an optimal configuration with minimal injury risk, by running a simulation of the imminent crash, and executing it in advance. All related topics such as mechanism design and decision-making can be investigated following the presented pipeline.

### 6.2 Geometries and arthrokinematics

A key point in our method is to reconfigure the skeletons along geometrically embedded trajectories. Model geometries are reconstructed via segmentation of tomography scans. Joints are not transformable at this moment, that is, we cannot reconfigure the segmented skeletons by specifying spatial orientation, e.g., rotation angle. For the dummy, components are joined by hinges, so trajectories are natively embedded in the structure. Inspired by the mechanical surrogates, it is necessary to prescribe the bone position associated with the given parameter, i.e., joint trajectories, when parameterizing skeletal movements of the FE-HBM.

The inferring trajectory from bone geometries is a classical topic. Orthopedic studies on associating knee arthrokinematics with bone contours date back to the 1840s (Weber and Weber, 1836). From the perspective of robotics, an individual rigid body has six DOFs, but the number of effective DOFs is much less. Due to constraints, only a few functional DOFs are allowed. Different from rigid mechanisms, soft connective tissues are flexible, and joints are usually over-constrained to enhance stability. The main types of constraints are the face-to-face constraint by articular surfaces and near-isometry constraints by muscles and ligaments. Energy dissipation tissues, e.g., intervertebral disc and meniscus, partly contribute as well.

The synovial joints have much higher mobility than the fibrous or cartilaginous joints. Mobility is of exceptional importance for repositioning. We have therefore especially emphasized these joints, i.e., the shoulder, elbow, hip, and knee, in this study. For synovial joints, a key characteristic is that bones are not physically connected but can move smoothly against each other along the articular surfaces covered by cartilage. The geometry of the articular surfaces defines the boundary of the movement. Geometrically compatible movements are restricted to a low-dimensional space. As elaborated, the shoulder, hip, and elbow are simplified as structurally similar hinges according to their function and morphology. These articulations are concave-to-convex type, e.g., acetabulum (concave) to femur head (convex), so we can intuitively infer arthrokinematics from the geometries. On the contrary, the knee is more complicated, as the articulation in the tibiofemoral joint is convex to convex, and there are two compartments. Conventional studies have incorporated the isometry assumption of the ligaments as additional constraints to complete the geometry–arthrokinematics problem (Wilson et al., 1998). The assumption ignores ligament flexibility and therefore leads to singularity in practice. Instead, we choose to decouple the constraints from articulation and explicitly parameterize the articulation-allowed motion subspace that holds the secondary variations led by ligament flexibility. Experimental studies have suggested that 3D tibiofemoral joint trajectory has in fact a 1-DOF (Gray et al., 2019). It implies that for an individual, the overall constraints from menisci, ligaments, and other connective tissues further restrict the arthrokinematics to a specific path inside the subspace, but additional physical clues beyond articulation geometries, e.g., meniscus elasticity and ligament orientation, are necessary. We deduce a geometrically neutral path among all the feasible choices discussed in Section 3. In the future, the solution can be updated if there is a way to better unify geometry and tissue flexibility. Besides, the framework can be easily implemented to any segmented femoral condyle and tibial plateau surfaces, leveraging the shape-informed registration technology (Tang et al., 2023). The algorithm can intuitively recognize the condylar and plateau regions of interest from the inferred correspondences and directly implement the steps thereafter.

The spine, as a combination of a series of cartilaginous joints, has a distinct structure when compared to the synovial joints. The bones are physically connected by cartilaginous tissues with a slight amount of movement available. Due to the absence of articulation, it is not intuitive to infer spine kinematics from its structural geometry. Besides, although the spine is very flexible, its motion is still highly limited when considering the large number of member segments. Different from the synovial joints for which we focus on and preserve the subject-specific geometries, we abstract the flexible spine as a 3D smooth curve according to the given pose parameters and devise a reserve operation to convert the curve back to structural instances in a geometrically and functionally compatible way.

In general, skeletons serve as a reconfigurable system, while pose parameters can specify the configuration of the system. Arthrokinematics offers a feasible and reasonable path for transition between configurations. The system can be dynamically reconfigured along the path, i.e., repositioned in an anatomically reasonable way.

### 6.3 Emulation *versus* simulation in deforming soft tissues

In this study, we emulate soft-tissue deformation according to skeleton movements with a series of numerical transformations instead of performing physics-based simulation as the pre-simulation approach does. The key motivation is to save time, as quasi-static pre-simulation is extremely time consuming. Another concern is that pre-simulation cannot guarantee correct joint trajectories. HBMs ignore a part of the fine-level details, especially secondary connective tissues, for computational efficiency and robustness. When pulling or moving parts like the limbs, the FE-HBM cannot be smoothly reconfigured as an intact human body due to the absence of these tissues. Instead, our method computes biofidelic arthrokinematics without demanding the structural integrity of the model and provides the necessary boundary conditions to transform soft tissues.

The proposed deforming method given in Section 4 is inspired by rigging, i.e., a substantial step in computer graphics (CG) for making animation. Reconfigurable virtual bones are rigged to 3D shapes. When the bones are transformed during skeletal movements in the human body, the rigged shape is transformed accordingly. Theoretically, rigging can be interpreted as a specific type of interpolation. Take linear skinning as an example. Movements associated with the virtual bone segments compose a group of bases, while a group of coefficients are assigned correspondingly to these points in space. The resultant movement of an individual point is a coefficient-weighted sum of the bases. If a point is close to a bone, the associated coefficient approaches 1, and the point tends to move rigidly with the bone.

We generally follow the interpolation idea. Tissues closely attached to underlying bones are transformed rigidly. The transitional part is interpolated via thin plate spline, with the associated bones as the landmarks. The interpolated variable is nodal displacement. The sliding-induced discontinuity in the displacement field makes the target capsule surface deviate from the naively interpolated shape. A part of the capsule nodes detaches from the originally interacted bone segment. We leverage a pseudo-contact algorithm inspired by the finite element solver to eliminate interpolation-induced penetrations and align the bone and capsule surfaces. Besides, as for an FE model–intended method, mesh regularity must be emphasized. Bones are not deformed when being rigidly transformed, therefore meshes are not affected, but the soft tissues experience a combination of stretching, compression, and distortion, which might severely deteriorate the quality of the involved elements. The basic idea is to keep the regular mesh topology on the boundaries. Nodal displacements are not consistent in interpolation and penetration elimination, so we introduce an optimization-based dynamic re-meshing step to redistribute the nodes while preserving the overall target shape. This improves the regularity of the elements on the boundaries and inside that are subsequently interpolated, and therefore the model generated by our method is natively ready for simulation on the FE structural level.

Emulation intends to mimic realistic physics, but it is not perfect after all. The current algorithm cannot preserve or control mass and volume of the transformed tissues. A feasible upgrade is to compute the deformation via pre-simulation with the derived arthrokinematics prescribed as the boundary condition. However, once FE simulation is incorporated, the computation will become time consuming. An alternative is to use a physics-based solver in computer graphics such as Taichi (Hu et al., 2019b), which is an intermediate between emulation and simulation that balances efficiency and accuracy. Some of these solvers support GPU acceleration and run much faster than conventional methods that are run on cluster.

### 6.4 Limitations and prospect

In this study, we propose a fast and biofidelic repositioning method for the FE-HBM compared to existing methods. However, repositioning a highly sophisticated human body model with a few given parameters is theoretically a non-trivial problem. Considering the complexity, time efficiency and accuracy are controversial and can hardly be satisfied at the same time. We propose a geometry-based estimation and hybrid deforming method for bone kinematics and soft-tissue deformation, respectively, to overcome the drawbacks in conventional methods, e.g., anatomically incorrect bone movements and severely distorted elements; however, there are still many simplifications and approximations. The derived arthrokinematics is fully geometry dependent. However, there are other physical constraints to consider in the real world, e.g., the four collateral and cruciate ligaments at the knee. This study has not considered the physiological and physical responses of the flexible connective tissues. In the future, it is promising to narrow down the geometrically compatible movements by incorporating the physical resistance of these tissues. Besides, the proposed emulation method is fundamentally interpolation based, and we have highlighted the consequent drawbacks and overviewed the next-step works in the last section.

Besides, in the near future, advances in computer graphics and distributed computing might promisingly reshape this field on both the software and hardware sides, as we have discussed in the last few sections.

## 7 Conclusion

We propose a fast and biofidelic toolbox to reposition FE-HBMs in a dummy-like manner. Given a group of pose parameters, e.g., rotation matrix and angle, this can rapidly change the HBM posture accordingly. In computation, skeletons are reconfigured along geometrically embedded trajectories, while soft tissues are transformed subsequently. On a technical level, we propose to estimate arthrokinematics of the synovial joints from the shape of the articular surfaces. The shoulder, elbow, and hip are approximated as structurally resembling hinges, i.e., spherical, cylindrical, and spherical, respectively. At the knee, the entire tibiofemoral trajectory is progressively retrieved by incrementally rotating the tibia. In each incremental step, we optimize for a helical axis, while rotation about the axis is instantaneously tangential at both the tibiofemoral compartments. After the skeletons are repositioned to a specific configuration, the soft tissues are deformed accordingly via interpolation. A morph–contact algorithm is suggested to tackle the sliding-induced singularity at the bone–capsule interface. It allows the capsule to wrap and slide along the repositioned skeletons in a physically reasonable way. Besides, an optimization-based refinement method is introduced to enhance element regularity by intuitively redistributing nodes without re-meshing.

The toolbox is very fast, as all incorporated transformations are simulation free. We implement the proposed toolbox to THUMS occupant and pedestrian models. The baseline models are repositioned to alternative postures, e.g., highly reclined occupant and the collision-avoiding pedestrian. The generated models are simulation ready and robust enough when exposed to high-speed impact loads.

## Data Availability

The raw data supporting the conclusion of this article will be made available by the authors, without undue reservation.
